# Application of Blue Filters Increases the Usefulness of Moreland Test in Anomaloscopic Color Vision Assessment for Blue–Green Color Range

**DOI:** 10.3390/ijerph18147654

**Published:** 2021-07-19

**Authors:** Krzysztof Piotr Michalak, Jacek Zabel, Jan Olszewski, Paulina Wojtyła-Buciora, Anna Przekoracka-Krawczyk

**Affiliations:** 1 Laboratory of Vision Science and Optometry, Faculty of Physics, Adam Mickiewicz University in Poznan, Uniwersytetu Poznanskiego Street 2, 61-614 Poznan, Poland; jacekz@post.pl (J.Z.); ania_pk@amu.edu.pl (A.P.-K.); 2 Department of Bionics and Experimental Medical Biology, Karol Marcinkowski University of Medical Sciences in Poznan, Parkowa Street 2, 60-775 Poznań, Poland; jolszewski@ump.edu.pl; 3 Department of Health Sciences, Calisia University-Kalisz, Nowy Swiat Street 4, 62-800 Kalisz, Poland; paulinawojtyla@gmail.com

**Keywords:** color vision, anomaloscope, Moreland, Rayleigh

## Abstract

The effect of blue light filters on the anomaloscopic examination was analyzed. Thirty subjects (18–43 y, 20 female, 10 male) without color vision disorders were examined in 4 filter conditions: no filter (F-0), Blue Control Hoya (F-BC), Med-1 JZO (F-Med1) and 450 Eschenbach (F-450). Both Rayleigh test (red–green axis) and Moreland test (blue–green axis) were performed. Application of F-BC filter shows negligible effect on color vision perception in both tests. Contrary to this, the application of strong F-450 filter causes significant shift in Moreland test towards tritanopy and the decrease in correlations of Moreland parameters with Rayleigh test parameters. The application of medium strong F-Med1 filter causes the slight shift in Moreland test towards the center of the Moreland scale and increases the Spearman correlations between Moreland and Rayleigh test parameters. This observation suggests that the about 15–40% reduction of blue diode intensity in the Moreland test may be beneficial in detecting mild changes in color vision perception in the blue-green axis and may improve its usefulness in evaluating the color vision perception disorders accompanying different illnesses, such as diabetes, glaucoma, neuritis optica, or cataract. The discussion concerning the modifications of Moreland test construction is also presented.

## 1. Introduction

Color vision disorders occur among the symptoms of numerous diseases of the human eye, including glaucoma [1,2], cataract [3,4], optic neuritis [5], and certain systemic diseases, such as diabetes mellitus [6,7]. Changes in color perception may affect the areas of red, green, and blue related to three types of cones (L, M, and S) in the retina, which are responsible for perception of three primary colors. Although color vision disorders are observed in the above diseases, they are not used to assess their severity. Such approach may be explained by several reasons. The first and the most important one is the scarcity of scientific research describing color vision deficits in particular diseases in a quantitative manner, the second is the scarcity of standardized diagnostic methods that would quantify color vision deficits, in particular in the green/blue range. Another issue is the inter-subject variability of color perception, which makes defining norms for healthy individuals ambiguous in certain cases.

Such variability might be related to:

(a) variability of primary structure of receptor proteins and minor peak shifts or other changes in their absorption spectra [8],
(b) variable number of cones of each type in the retina and the macula [9],
(c) differences in the structure or functioning of the retina’s neuronal network, which codes information concerning the activity of each cone type and transfers the color information to the central nervous system [10],
(d) differences in color perception at the brain cortex level.

Particularly, aspects (c) and (d) above have not been well-researched so far in the literature.

Despite these limitations, developing a methodology for quantifying color perception and analyzing changes in color perception in individual diseases is an important direction of research. In the studies carried out to date in our laboratory, the researchers have assessed the anomaloscopic test, which enables quantitative assessment of color perception in the red/green (Rayleigh test) and green/blue (Moreland test) [11] axes. The Rayleigh test is well standardized and the results are comparable. This test is primarily used to quantify deficiencies of red–green perception. In contrast, the Moreland test has been poorly studied and is practically not used for diagnostic purposes in clinical practice. The Rayleigh test is based on a comparison of a subjective impression of the color yellow (produced by mixing green and red) with a standardized yellow color. As a result of the measurement procedure, border values of light intensity ratio are defined for the red and the green diodes (R_1_ and R_2_). The R value can range between 0 (100% green diode) and 73 (100% red diode) anomaloscope units [12]. The diodes allow the subject to perceive the test color as being identical to the reference color. An additional parameter is the brightness of the yellow reference color at the color adjustment borders (Y_1_, Y_2_). Similarly, the Moreland test is based on comparing the subjective impression of the cyan in the test field (resulting from mixing the light emitted by green and blue diodes) with the standard cyan diode. The resulting measurement values are the border values of intensity for the blue and the green diode, which give make the subject perceive the test color as being identical to the standard cyan color (M_1_ and M_2_). Another test outcome is the brightness of the reference cyan color at the time of shade equalization (C_1_, C_2_). The M value can range between 0 (100% green diode) and 100 (100% blue diode) anomaloscope units.

Recently, Zabel et al. [11] presented the results of anomaloscopic measurements of Polish subjects aged below 45 years without color vision disorders, as determined by the HRR method. The subjects were free from eye diseases that might affect changes in color perception. The Rayleigh test results obtained by the research team, as compared to the standards recommended by the anomaloscope manufacturer, were slightly shifted towards protanomaly. The recommended standard according to the anomaloscope user’s manual is 34–46 units [12], while the results obtained in the study (±2 std) were 35.4–51.3 for females and 36.8–54.8 for males. Additionally, it was observed that the distribution of the R_2_ parameter was characterized by two maxima with a minimum between the peaks located around the value of 50. Values above 50 were observed mainly in male subjects. Based on the above observations, it was suggested that approximately 20% of males should be classified as a subtle protanomaly group. The result may be related to the fact that opsin (protein) is encoded on the X chromosome and men have only one copy of this gene. However, such an assumption poses a problem in determining a norm for the male population. The norms suggested above, however, include 2 std around the mean (95% of the population) and therefore the majority of the subtle protanomaly peak is included in the norm.

The results obtained for the Moreland test differed significantly from the norm proposed by the anomaloscope manufacturer. It was observed that the fitting range was significantly wider (M_C_ = M_2_ − M_1_) and considerable inter-subject variability for the border parameters M_1_ and M_2_ was also noticeable. In particular, such variability was observed in case of the M_1_ parameter. For this reason, the Moreland test in its current version seems to have little value for quantification of color vision disorders in the green/blue axis.

In the majority of subjects, the impression of blue was dominant in a wide range of the proportion of green and blue diode intensities. Thus, one of the possible explanations of the large variability of the Moreland test results obtained in this study was too high intensity of the blue diode, which dominated over the green. In order to investigate this hypothesis in the present work, the Rayleigh and Moreland test were administered using additional lenses which partially filtered blue light. Among the available filters, the researchers selected three: Blue Control (F-BC) by Hoya, Med. 1 filter (F-Med1) manufactured by JZO and F-450 by Eschenbach. The results obtained using each of the three filters were compared against test results without any filter (F-0). It was expected that reducing the intensity of the blue diode should result in a reduction of the extremely high value of the M_2_ parameter and a reduction of the variability of M_1_ values. Consequently, a reduced width of the matching range (M_C_) could contribute to a greater diagnostic value of the test with the use of a specific filter.

Anomaloscopic examination enables the assessment of three aspects of color vision:

1. The first aspect is the assessment of the relative subjective brightness of the tested primary colors red, green, and blue, which correspond to the maxima of absorption spectra of subsequent cones L, M, and S.
2. The second one is the ability to perceive changes in the different shades and tones, i.e., neighboring RGB colors may be perceived either as the same or slightly different in quality.
3. The third aspect is quite similar to the first one but, nevertheless, it seems reasonable to distinguish between them. It is the general ability to perceive the given color being understood as the sensitivity of the given retinal cones to the given wavelength.

The assessment of relative subjective brightness may be assessed based on the Y = fn(R) relation in the Rayleigh test and the C = fn(M) relation in the Moreland test, where Y is the luminance of the yellow reference diode as a function of R (the proportion of red to green), and C is the luminance of the cyan reference diode as a function of M (the proportion of blue to green). In the case of the Moreland test, the sensitivity to blue light is generally lower than sensitivity to green light and therefore the slope (a_M_ = (C_2_ − C_1_)/(M_2_ − M_1_) is positive and approximately equal to 1. For example, if the evaluated individual perceives 436 nm blue light as relatively darker than it is perceived by others, then the a_M_ slope for the C = fn(M) relation is higher. As the M setting is increased (i.e., more green is added and blue is reduced), the perceived brightness increases more rapidly. In the Rayleigh test, red is perceived as a relatively darker color than green, therefore, a_R_ is negative. If a given subject perceives red as being darker (in comparison to other individuals’ perception), then, as the proportion of red is increased, the perceived brightness decreases at a higher rate and the a_R_ ratio becomes more negative.

The assessment of the ability to perceive subjective changes in the color tones between blue and green (sea color/cyan/aquamarine/turquoise/olive) may be defined as the width of the matching range (M_W_ = M_2_ − M_1_, R_W_ = R_2_ − R_1_). The more narrow the color matching range, the more efficiently the adjacent shades and tones are perceived.

As for the third aspect, i.e., the overall ability to perceive colors, a reduced ability to perceive a particular primary color does not necessarily mean that the color will be perceived as darker. For example, a reduced ability to perceive red will be manifested as a shift of the $R_{C}=\frac{R_{1}+R_{2}}{2}$ parameter to the right. It means that a more red diode must be added for the resulting color (i.e., red mixed with green) to be perceived as identical to the reference yellow.

However, this does not necessarily mean that exemplary red will be perceived by the subject as darker (a higher slope in the Y = fn(R) relation). The overall ability to perceive a particular color will be manifested mainly when it is mixed with one of other primary colors. The differentiation between the perceived color brightness and the ability to perceive a given color has not been well researched to date and has not been discussed in the literature. In general, it is more complex to analyze than the two previously mentioned aspects.

It should be emphasized that if anomaloscope is to be used to detect subtle changes in color perception (due to disease processes) rather than to detect major deficits (related to genetic defects), the three aspects must be understood and analyzed separately. In addition, a perception disorder related to a particular disease may affect only one of the above aspects. So far, anomaloscopic examinations have been mainly based on the Rayleigh test administered in order to assess vision of the red color, i.e., the need to increase the amount of red light during the test in order to achieve an impression of yellow in the test field that matches the color in the reference field. It is manifested by a shift of R_2_ and R_C_ to the right and widening of the matching range (R_W_). The Y parameter of the Y = fn(R) function has not been used to date to assess color vision disorders resulting from particular disease processes.

In this article, apart from the assessment of blue light filters usability, an analytical proposal for the application of the above parameters is described. The analyzed parameters may be obtained from anomaloscopic examinations using Rayleigh and Moreland tests.

## 2. Methods

### 2.1. Group

Inclusion criteria were: no general or ocular diseases, and no regular medication used, normal visual acuity (VA 0.8 or better, 0.1 logMAR or better), normal color vision as determined by HRR test. The subjects were first screened by ophthalmologists, who checked their VA, ocular health (anterior and posterior segments), and then subsequently evaluated during optometric examination, including eye dominance test and confirmation of normal color vision using the HRR test.

Thirty subjects were selected to the experiment, aged 18–43 years, including 20 females and 10 males. HMC MR Oculus type 47700 anomaloscope was used for measurement of color perception with filters. This part of the research was conducted in a dimly lit room and each subject adapted to the test conditions for at least 10 min prior to the assessment. The test was performed in the dominant eye and the order of using the filters was randomized. The Rayleigh and Moreland tests were conducted in the manual mode. The detailed algorithm of the study is presented in Appendix A.

All tests were administered in the ‘*Zabel sc.*’ Ophthalmology Consultation Center (*Poradnia Okulistyczna Zabel sc*.) in Piła, Poland. The study was carried out in accordance with the Helsinki Declaration following approval from the UMP bioethics committee at the Karol Marcinkowski Medical University of Poznan.

### 2.2. Procedure

Each of the subjects underwent the Rayleigh and Moreland tests four times, i.e., using three types of filters (F-BC, F-Med1, F-450) plus no filter (F-0). The border matching values have been recorded (R_1_, R_2_, M_1_, M_2_) together with the corresponding brightness levels of the reference field (Y_1_, Y_2_, C_1_, and C_2_). Based on the above values, the following parameters were calculated:

$$R_{C}=\frac{R_{1}+R_{2}}{2}$$

R_W_ = R_2_ − R_1_

$$a_{R}=\frac{Y_{2}-Y_{1}}{R_{2}-R_{1}}$$

$$M_{C}=\frac{M_{1}+M_{2}}{2}$$

M_W_ = M_2_ − M_1_

$$a_{M}=\frac{C_{2}-C_{1}}{M_{2}-M_{1}}$$

Initially, it was investigated which of the above parameters changed significantly following the application of a particular type of filter. The vast majority of the analyzed variables distributions were not normal in the Shapiro–Wilk test and, therefore, statistical analyses were performed using the Wilcoxon test and the Spearman’s rank-order correlation.

Subsequently, the correlations between the Rayleigh and Moreland test results were examined in order to research the impact of red color perception on the ability to perceive colors in the green–blue axis and to assess the impact of blue perception on color perception in the red–green axis.

**Figure 1:**
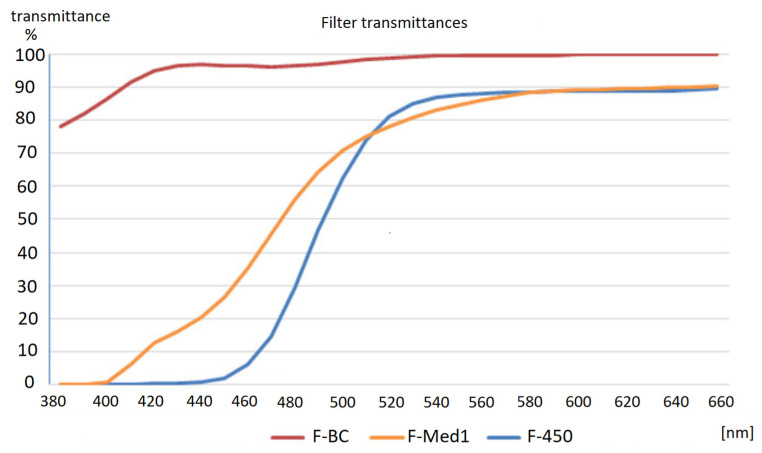
Characteristics of the filters used in this study: F-450 (Eschenbach), F-BC Blue Control (Hoya) and F-Med1 (JZO).

The first of the filters used in the current study was Hoya’s Blue Control which reduces the 436 nm wavelength by 4%, 480 nm by 3% and 490 nm by 3% (Table 1, Figure 1). It is dedicated to users of digital devices in order to provide protection against adverse consequences of excessive blue light exposure. The filter provides a relatively low reduction of blue light and does not significantly modify color perception.

The second filter (Figure 1) was JZO’s Med. 1 which reduces the 436 nm wavelength by 84%. The filter’s characteristics are presented in Table 1. Its transmittance for 480 nm and 490 nm wavelengths is 56% and 64%, respectively.

The last filter (Figure 1) used was Eschenbach’s F-450 edge filter which virtually eliminates the 436 nm wavelength and simulates the symptoms of severe tritanopia during the test.

Comparison of transmittance levels for each filter type, as per individual light wavelengths, is presented in Table 1.

## 3. Results

### 3.1. Basic Results of the Moreland Test

**Figure 2:**
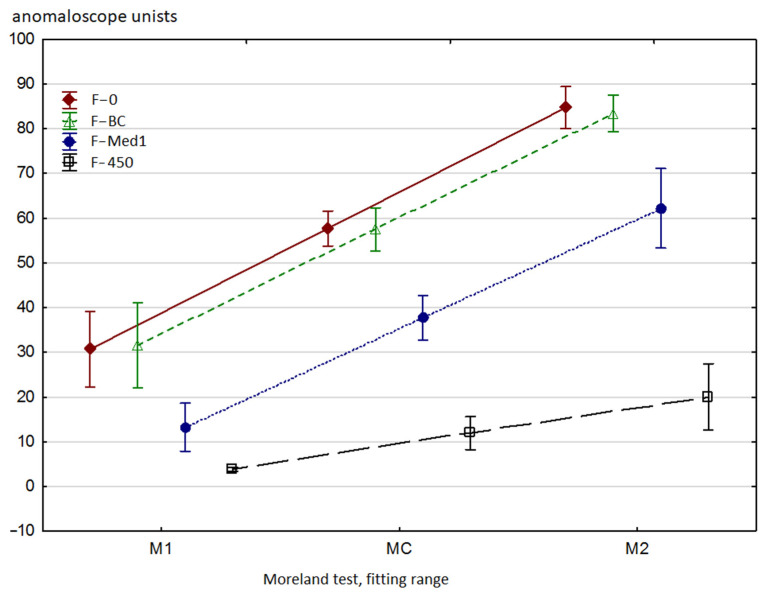
The results for M_1_, M_C_ and M_2_ in the Moreland test for each type of applied filters (mean/mean standard error). The anomaloscope units refer to the blue–green diode proportion in the test field: 0 au: 100% blue and 0% green; 100 au: 100% blue and 0% green. The Blue Control filter did not significantly affect the results due to its minimum absorption of blue light. Application of the F-Med1 filter significantly decreased M_1_ and M_2_ but did not considerably reduce the width of the matching range. The oscillation range of the M_1_ variable was reduced but, conversely, the variability of M_2_ increased. The use of the F-450 filter reduced M_1_ and M_2_ values to very low levels. Matching the test field color to the reference field color was possible only provided that a high intensity of blue diode light was set. Thus, the test results were as expected for patients with tritanopia. All the differences between the filter types were statistically significant for M_1_, M_2_ and M_C_, except for the F-0 vs. F-BC comparison.

As expected, reduced intensity of blue light (436 nm) decreased the values of M_1_ and M_2_ matching parameters (see Figure 2). The efficacy of the Blue Control filter was too low to induce any statistically significant changes. The F-Med1 filter, which reduced the amount of 436 nm light down to 16%, caused a decrease in M_1_ (from 30 to 13) and M_2_ (from 85 to 62). The M_C_ parameter has also decreased accordingly. M_W_, which describes the width of the matching range, was significantly reduced only following the application of the F-450 filter, which almost completely stops blue light (see Table 2).

### 3.2. Color Matching C = fn(M) Slope

**Figure 3:**
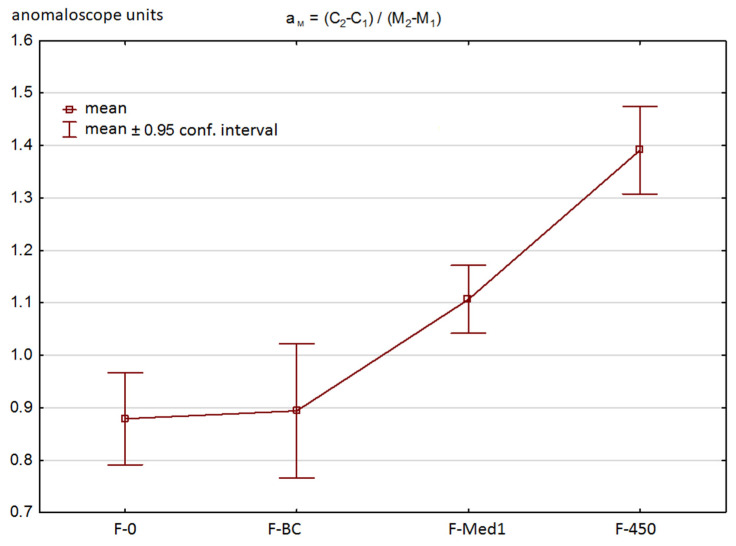
Values of the slope (a_M_) for C = fn(M) matching (mean/mean standard error). As the amount of blue light is reduced by a filter, the value of the slope ratio increases. This reflects the reduced impression of perceived brightness of blue when a filter is introduced. Except for the F-0 vs. F-BC comparison, other differences had high statistical significance (*p* < 0.001) in the Wilcoxon test.

The slope (a_M_) of the C = fn(M) color matching function reflects the relative difference of brightness between blue and green. The steeper the slope of the above relationship (i.e., the higher the value of a_M_), the more the green color will be perceived as relatively brighter, as compared to blue. When the green to blue proportion is increased, the perception of brightness will increase more rapidly, which is manifested as an increase of luminance of the cyan field being matched (480 nm) to the reference field. The obtained a_M_ values for each of the filter types are presented in Figure 3.

Subsequently, the correlations between the perceived brightness (a_M_, aspect 1, see Introduction) and other parameters of the Moreland test were studied. The main goal of the research team was to identify to what extent the parameter was correlated to the values of M_1_, M_2_, and M_C_ (i.e., the ability to perceive blue and green, aspect 3). The results are presented collectively in Table 3.

In the F-0 test, there was a weak negative correlation for M_2_ and M_W_. This means that the individuals who perceive blue as being relatively darker (high a_M_) exhibit slightly lower values of M_W_ and M_2_, which also means that the general perception of blue is relatively poorer as compared to green. However, the correlation is poor, which indicates that the relative perception of blue as being darker may only partially be explained by its lower perception. Similar results were obtained for the F-BC and F-Med1 filters, whereas the F-Med1 filter improved statistical significance of the correlation for M_W._ Introduction of the F-Med1 filter shifted the Moreland test results closer to the central area of the test range (see Figure 2). Thus, the results reflect the differences between green and blue color perception somewhat better, which might have been the reason for a slightly stronger correlation between M_W_ and a_M_. Meanwhile, the F-450 filter cut out so much light coming from the blue diode that the comparison of blue perception vs. green became problematic and all correlations in this regard disappeared.

### 3.3. The Impact of Filters on the Rayleigh Test Results

The use of the tested blue light filters could have had a minor impact on the obtained values of the Rayleigh test parameters due to the slight reduction of the 546 nm green light used in the Rayleigh test (see Table 1). However, the effect of filtering is also possible due to the fact that the L (red) and M (green) cones are also, to some extent, sensitive to blue light.

**Figure 4:**
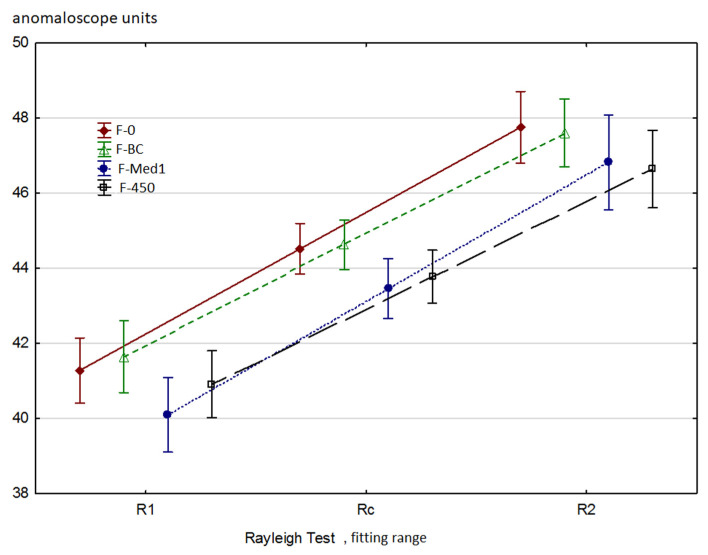
The impact of filters on the Rayleigh test results. The anomaloscope units refer to the red-green diode proportion in the test field: 0 au: 100% red and 0% green; 73 au: 100% red and 0% green. A certain reduction in R_1_ and R_2_ values is noticeable following the application of the F-Med1 and the F-450 filters. Statistical significance of the differences observed in the Wilcoxon test is presented in Table 4. R_1_ results are presented top-right while R_2_ results are to be found bottom-left.

The results obtained by the researchers are presented in Figure 4. Table 4 presents the statistical significances of the differences between the various filters for parameters R_1_ and R_2_. A slight decrease in the values of the R_1_ and R_2_ parameters can be observed when using the F-Med1 and F-450 filters. The obtained results indicate a slightly weaker perception of green, which is consistent with the characteristics of the filters used as they slightly absorb green light (546 nm) used in the Rayleigh test. Interestingly, the use filters did not significantly affect the values of the R_W_ and a_R_ parameters. Cutting out a small portion of the green light used in the Rayleigh test did not affect the quality of distinguishing similar color tones in the lemon-yellow–orange area. Regarding the a_R_ parameter, there is a noticeable tendency for a_R_ to decrease (i.e., become more negative) with all filter types when compared to the test without any filter (NF). This is consistent with the fact that green light intensity is slightly lower. When analyzing the a_R_ parameter, it should be noted that the width of the color matching area (R_W_) is narrower in the Rayleigh test than in the Moreland test. Therefore, the accuracy of estimating the values of R_1_, Y_1_, R_2_, and Y_2_ may significantly affect the accuracy of calculating the value of a_R_.

### 3.4. Correlations between the Rayleigh and the Moreland Tests

Perception of colors, their tones and shades by the human eye and the central nervous system is a complex process involving a number of aspects which have not been thoroughly researched to date. Perception of the entire scale of color tones depends on the relative proportion of activity of only three types of cones. Therefore, it seems justified to claim that perception of various color tones and differentiation of shades depends on the mutual proportion of the extent to which all three cone types are stimulated. Therefore, it may be expected that there is a certain correlation between the results of the Rayleigh and Moreland tests. For that reason, the results of both tests were compared and Spearman’s correlation factors were defined for the four blue light filtering scenarios. The results are presented collectively in Table 5.

## 4. Discussion

**Figure 5:**
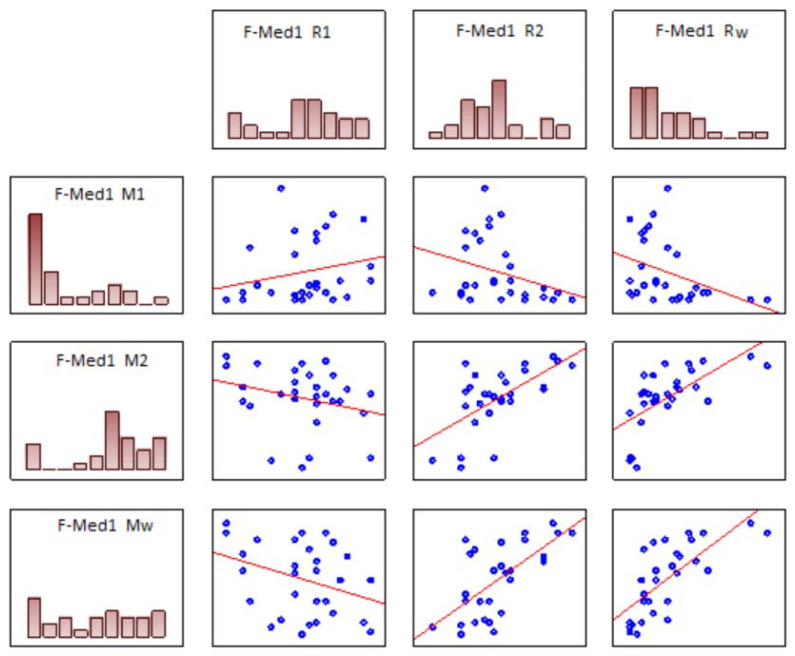
Selected correlations between the Moreland and the Rayleigh tests for the F-Med1 filter. Detailed correlations are listed in Table 5. Notice the particularly strong correlation between R_W_ and M_1_ (negative), as well as M_2_ and M_W_ (positive). Efficient perception of color shades in the lemon-orange range in the Rayleigh test was strongly correlated with efficient perception of shades in the blue-cyan-green axis. Such effect is best visible following the reduction of the amount of blue light with the F-Med1 filter (down to approx. 16%).

The data presented in Table 5 and in Figure 5 indicates that the results of the Moreland test are significantly correlated mainly with the values of R_W_ and, to a lesser extent, with R_1_ and R_2_. R_W_ is related to the ability to distinguish color tones in the lemon-yellow-orange axis. It is noticeable that a good ability to differentiate color tones in the Rayleigh test also allowed the subjects to better differentiate between color tones in the green–blue axis. In the case of F-0 tests, such effect was noticeable primarily as an increase in M_1_ values i.e., the parameter, which shows significant inter-subject variability. M_1_ was also correlated to R_1_, which leads to the conclusion that the correlation mainly depends on the quality of green perception. Efficient perception of green (i.e., high R_1_ value) causes statistically higher values of M_1_ and more narrow matching ranges (M_C_) in the Moreland test.

When blue filters were applied, the relation was somewhat different. The correlation between R_W_ and other Moreland test parameters was still observable while the relationship with R_1_/R_2_ was altered. The correlation to R_1_ diminished in case of the F-BC filter, a strong correlation to R_2_ occurred for the F-Med1 filter, while the correlations both to R_1_ and R_2_ were no longer noticeable in case of the F-450 filter.

It should be noted that, in case of the F-Med1 filter, the correlations reached values between 0.6 and 0.8, which makes the results of both tests significantly aligned.

Since R_2_ is mainly related to the quality of red perception, it is reasonable to conclude that a reduction of blue light and, to a lesser extent, the amount of green light, when the F-Med1 filter is applied, increases and emphasizes the role of L cones and the L-M information in the perception of blue and green light as well as color tones in the green-cyan-blue axis. Proficiency in differentiating color tones in the lemon-yellow–orange axis increases the competence in differentiating between the color tones in the blue-cyan-green axis.

In case of F-Med1, there is also a clear negative correlation of a_M_ to R_2_ and to R_C_. This means that low values of R_2_ and R_C_ (i.e., better red perception) is associated with higher a_M_ values (i.e., blue is relatively darker or green is brighter). It can be carefully concluded that higher individual number of red cones in the retina is correlated with lower number of blue cones rather than with the green ones and blue light reduction magnifies this effect.

It is arguable that a decrease in luminance of the blue diode in the Moreland test enhances the correlations between the tests in terms of green perception which is common for both test types. Such result is another indication that the intensity of light emitted by the blue diode in the Moreland test is too high and reducing it by 15–40% percent may be beneficial in the context of detecting certain abnormalities. This may be applicable especially in case of decreased green perception ability.

An important observation was also that the correlations between R_C_ and M_C_ were low while the correlations between R_1_, R_2_, M_1_, and M_2_ were high, which indicates that the limit parameters (R_1_, R_2_, M_1_, and M_2_) should be considered independently from one another and that calculation of mean values makes the related information less convincing. R_C_ and M_C_ are significant in cases of more pronounced color vision deficits but they are less meaningful when more subtle differences are considered.

The results discussed above indicate that there is a significant and unused potential of anomaloscopic examination in terms of quantitative analysis of subtle inter-subject differences in terms of color perception. The same is also true for this examination in the context of quantitative assessment of deterioration in the perception of certain colors in the course of various disease processes affecting the visual and nervous systems. The application of blue light filters presented herein suggests that there is a significant potential for further analyses and new evaluation parameters in cases of color vison disorders due to an underlying disease process.

One of the main difficulties, which hinders the acquisition of valuable results related to disease severity in the context of color vision, is inter-subject variability. The application of filters, including those with different parameters such as those reducing red or green light, may provide additional parameters which may be less related to the inter-subject variability and may serve as better indicators of the characteristics of certain disease processes. Further analyses may also shed some light on the theory of opposing colors, the characteristics of color coding in the retina as well as color perception by the central nervous system.

It also seems justified to modify the Moreland test so that the difference between the wavelengths of the green diode (490 nm) and the cyan reference diode (480 nm) becomes more significant. Thus, the color matching area in the Moreland test could be narrowed down, which may facilitate more precise analysis. However, this shall require redesigning of the anomaloscope and a revision of the reference ranges for a new set of diodes used in the green–blue axis test. It might also be interesting to design a test able to compare blue directly to red by means of comparison of the red–blue color mix in the test field with a reference violet diode. Valuable results may also be expected from the application of a filter characterized by significantly different transmittance for 480 nm and 490 nm wavelengths, as this would allow test subjects to better differentiate between the green and cyan diodes (with the design of anomaloscope remaining as is). The filter suggested here could possibly have transmittance levels of 15–40% for 436 nm, 50–80% for 480 nm, and 95–100% for 490 nm. Application of such filter might significantly reduce the excessive width of the matching range (M_C_) in the Moreland test and could facilitate more efficient diagnosis of blue color vision deficits.

## 5. Conclusions

Reduction of blue diode intensity in the Moreland anomaloscope test by about 15–40% may be beneficial in detecting mild changes in color vision perception in the blue-green axis and may improve its usefulness in evaluating the color vision perception disorders accompanying different illnesses.

## Figures and Tables

**Table 1:** Transmittance of filters used in the study in relation to wavelengths emitted by anomaloscope diodes.

Filter’s Light Transmittance	436 nm (Blue Diode)	480 nm (Cyan Diode)	490 nm (Green Diode, Moreland)	546 nm (Green Diode, Rayleigh)
F-BC	96%	97%	97%	99.7%
F-Med1	16%	56%	64%	84%
F-450	0.3%	29%	47%	87%

**Table 2:** The results for the analyzed parameters (mean ± standard deviation) and statistical significance of the differences in the Wilcoxon test as compared to the results in the preceding columns; no—no significance, +—*p* < 0.05, *—*p* < 0.01, **—*p* < 0.001.

Parameter	No Filter (F-0)	F-BC (F-0)	F-Med1 (F-0, F-BC)	F-450 (F-0, F-BC, F-Med1)
R_1_	41.3 ± 2.3	41.6 ± 2.6 (no)	40.1 ± 2.7 (+, **)	40.9 ± 2.4 (no, +, +)
R_2_	47.7 ± 2.5	47.6 ± 2.4 (no)	46.8 ± 3.4 (no, +)	46.6 ± 2.8 (*, *, no)
R_W_	6.5 ± 3.3	5.9 ± 3.5 (no)	6.7 ± 4.3 (no, no)	5.7 ± 3.5 (no, no, no)
R_C_	44.5 ± 1.8	44.6 ± 1.8 (no)	43.5 ± 2.1 (*, *)	43.8 ± 1.9 (+, *, no)
a_R_	−0.12 ± 0.79	−0.38 ± 0.34 (no)	−0.37 ± 0.38 (no. no)	−0.39 ± 0.51 (no. no. no)
M_1_	30.7 ± 22.6	31.6 ± 25.5 (no)	13.3 ± 14.1 (*, *)	3.9 ± 1.2 (*, *, *)
M_2_	84.7 ± 12.5	83.4 ± 11.0 (no)	62.2 ± 23.3 (*, **)	20.1 ± 19.9 (*, *, **)
M_W_	54.0 ± 30	51.8 ± 29.6 (no)	48.9 ± 28.6 (no, no)	16.2 ± 20.1 (*, *, **)
M_C_	57.7 ± 10.4	57.5 ± 12.9 (no)	37.7 ± 13.0 (*, **)	12.0 ± 9.8 (*, *, **)
a_M_	0.88 ± 0.23	0.89 ± 0.34 (no)	1.1 ± 0.17 (*, **)	1.39 ± 0.22 (*, *, **)

**Table 3:** Comparison of Spearman’s rank correlation coefficient (r) for a_M_ vs. other Moreland test parameters. The correlations were weak or none, which means that the aspect of perceived color brightness is essentially different from the general ability to perceive the blue and green colors. *—*p* < 0.05.

Moreland Test Parameter	Filter-0	F-BC	F-Med. 1	F-450
M_1_	r = 0.244	r = 0.071	r = 0.171	r = −0.013
M_C_	r = 0.089	r = −0.012	r = −0.187	r = 0.049
M_2_	r = −0.374 *	r = −0.292	r = −0.369 *	r = 0.054
M_W_	r = −0.315	r = −0.112	r = −0.406 *	r = 0.042

**Table 4:** Statistical significance of the differences in the Wilcoxon test for R_1_ and R_2_, as per filter types (see Table 2 and Figure 4). R_1_ results are presented top-right while R_2_ results are to be found bottom-left. The effect of filtering on both parameters is noticeable, while there is no impact on R_W_ (i.e., the ability to perceive color tones between lemon and orange).

R_1_ (Top/Right) R_2_ (Bottom/Left)	F-0	F-BC	F-Med1	F-450
F-0	---	*p* = 0.304	*p* = 0.010 **	*p* = 0.242
F-BC	*p* = 0.590	---	*p* < 0.001 ***	*p* = 0.012 *
F-Med. 1	*p* = 0.062	*p* = 0.044 *	---	*p* = 0.038 *
F-450	*p* = 0.007 **	*p* = 0.004 **	*p* = 0.959	---

*—*p* < 0.05, **—*p* < 0.01, ***—*p* < 0.001.

**Table 5:** Spearman’s rank correlations between the parameters of the Rayleigh and Moreland tests. It should particularly be noted that there is a strong correlation between R_W_ and most of the Moreland test parameters for each of the filter types. A narrow matching range (R_W_), i.e., good perception of shades in the lemon–orange range, was correlated to high M_1_, low M_2_ and low M_W_, which means that efficient perception of color shades in both tests was correlated. The strongest correlations (|r| > 0.5) were observed in case of the F-Med1 filter and were presented in detail in Figure 5. *—*p* < 0.05, **—*p* < 0.01 (marked with bold).

**F-0**					
r	R_1_	R_2_	R_C_	R_W_	a_R_
M_1_	0.458 *	−0.099	0.14	−0.459 *	0.109
M_2_	−0.094	0.134	0.108	0.246	0.125
M_C_	0.415 *	−0.096	0.146	−0.404 *	0.133
M_W_	−0.355	0.12	−0.05	0.420 *	0.071
a_M_	0.121	0.205	0.229	0.134	0.125
**F-BC**					
r	R_1_	R_2_	R_C_	R_W_	a_R_
M_1_	0.149	−0.261	−0.001	−0.406 *	−0.116
M_2_	−0.057	0.460 *	0.205	0.445 *	0.252
M_C_	0.162	−0.068	0.099	−0.266	−0.044
M_W_	−0.156	0.359	0.064	0.480 *	0.113
a_M_	0.039	−0.344	−0.111	−0.285	−0.077
**F-Med. 1**					
r	R_1_	R_2_	R_C_	R_W_	a_R_
M_1_	0.347	−0.34	−0.069	−0.477 *	−0.264
M_2_	−0.255	0.583 **	0.295	0.712 **	0.294
M_C_	−0.11	0.24	0.117	0.3	0.125
M_W_	−0.303	0.640 **	0.294	0.793 **	0.338
a_M_	−0.271	−0.431 *	−0.480 *	−0.261	−0.184
**F-450**					
r	R_1_	R_2_	R_C_	R_W_	a_R_
M_1_	0.105	−0.348	−0.131	−0.363 *	−0.103
M_2_	−0.183	0.29	0.093	0.468 *	0.032
M_C_	−0.165	0.255	0.11	0.437 *	−0.013
M_W_	−0.173	0.307	0.091	0.480 *	0.066
a_M_	0.102	0.0147	−0.02	−0.152	−0.147

## Abbreviations

R_1_/R_C_/R_2_—left border/center/right border of the fitting range in the Rayleigh anomaloscope test (red–green axis); R_W_ = R_2_ − R_1_—width of the fitting range in the Rayleigh anomaloscope test; M_1_/M_C_/M_2_—left border/center/right border of the fitting range in the Moreland anomaloscope test (blue–green axis); M_W_ = M_2_ − M_1_—width of the fitting range in the Moreland anomaloscope test; a_R_—the slope of the relation Yellow Brightness vs. Red–Green proportion in the Rayleigh test; a_M_—the slope of the relation Cyan Brightness vs. Blue–Green proportion in the Moreland test.

## Appendix A

**HRR test:** The HRR test was performed monocularly in the room with intensive artificial light. If the person had problem at anyone level of the test, he/she was excluded from the further experiment. Next, the subject who fulfilled correctly HRR test, was send for optometric examination (visual acuity and refractive error measurements).

**Anomaloscope test:** This test was performed after the HRR and optometric examinations. HMC Anomaloscope MR type 47700 (Oculus) was used. The non-tested eye was covered with black patch. The order of eyes was counterbalanced. The ilumination in the room was low. The persons were adopted to the room ilumination by 10 min. Before the main test, the demo test was performed in order to explain the measurement procedure.

Both Rayleigh and Moreland tests were performed manually. Firstly, the Rayleigh test was carried out. The upper and lower limit has been found using the staircase method. The initial value of the reference field was 40 units. The measurement of higher limit (R_2_) preceeded lower limit test (R_1_). For R_2_, the reference field was increased by 5 up to reaching the lack of possibility of adjusting the reference and test field. Next, the actual range for R_2_ was divided by half in order to tighten the range of R_2_. This procedure was repeated up to reaching the measurement limit of the device equal to 0.5. Next, R_1_ was measured in simiar way. The R_1_ and R_2_ values were measured with precision 0.5. After the first eye examination, 2–3 min break was performed and the second eye was measured with Rayleigh test.

After the next 2–3 min break, the Moreland test started. The initial reference value was 50 units. The measurement of M_2_ preceeded M_1_. For M_2_, the reference value increased by 10 up to reaching the lack of possibility of adjusting the reference and test field. Next, the actual range for M_2_ was divided by half in order to tighten its range. The procedure was repeated up to reaching the precision of M_2_ equal to 1 unit. After one eye was measured, 2–3 min break was performed and the second eye was measured.
